# Current perspectives in sudden unexpected death in epilepsy (SUDEP): epidemiology, research approaches and pathways to prevention

**DOI:** 10.1186/s42466-026-00480-w

**Published:** 2026-03-24

**Authors:** Catrin Mann, Susanne Schubert-Bast, Felix Rosenow, Adam Strzelczyk

**Affiliations:** 1 Department of Neurology, Epilepsy Center Frankfurt Rhine-Main, Goethe University Frankfurt, University Medicine Frankfurt, Theodor-Stern-Kai 7, Frankfurt am Main, 60596 Germany; 2 Department of Pediatrics, Pediatric Epileptology, Goethe-University Frankfurt, University Medicine Frankfurt, Frankfurt am Main, Germany

**Keywords:** Seizures, Mortality, Risk, Respiration, Antiseizure medication, Sleep

## Abstract

People with epilepsy (PWE) are affected not only by the unpredictability of seizures, the risk of accidents, stigma, and comorbidities, but also by increased mortality. The most common directly epilepsy‑associated cause of death is sudden unexpected death in epilepsy patients (SUDEP). Across all PWE, SUDEP affects approximately 1 in 1,000 patients per year; depending on epilepsy severity, the annual SUDEP rate can exceed 10 per 1,000 patient years. Because many PWE live with the disorder for several decades, the cumulative SUDEP risk amounts to an average lifetime risk of 5–20%, rendering SUDEP a relevant contributor to mortality in PWE; however, considering its comparatively low annual incidence, SUDEP research remains challenging. Established risk factors and associated patient characteristics include frequent bilateral tonic–clonic seizures (BTCS) -especially when nocturnal-, living alone, long duration of epilepsy, and drug-resistant epilepsy. There are few observations in humans regarding the pathophysiological mechanisms underlying SUDEP, supplemented by findings from registries, animal models, and theoretical considerations. In the typical SUDEP cascade, a pathologically impaired arousal following a preceding, often nocturnal, BTCS appears to precipitate apnea and consequent bradycardia, progressing to fatal asystole. Various individual vulnerability factors contribute to this cascade. However, many aspects remain poorly understood. Two large, recently published prospective cohort studies have contributed valuable insights into biomarkers of SUDEP. Perisylvian epilepsies were identified as risk factors; furthermore, findings suggest dysfunctional brainstem respiratory regulation and impaired sleep homeostasis as potential mechanisms contributing to SUDEP. Nevertheless, on an individual level, it remains poorly understood why some patients die in the context of their first ever epileptic seizure, while others survive hundreds of BTCS. This narrative review provides an overview of the current state of SUDEP research and information on preventive measures used today, and delineates prospective directions for future investigation and prevention.

## Introduction

People with epilepsy (PWE) face an approximately threefold increased risk of premature mortality [22]. The risk of sudden and unexpected death is increased by up to approximately 24-fold compared to the general population [20]. Sudden unexpected death in epilepsy patients (SUDEP) is the leading cause of directly epilepsy-related mortality in PWE, accounting for a substantial proportion of the excess mortality observed across age groups and clinical settings. Epidemiological estimates indicate that SUDEP is responsible for up to one third of premature mortality in PWE [66], underscoring its clinical and public health relevance. The age‑specific incidence of SUDEP peaks in the third and fourth decades of life. In developmental and epileptic encephalopathies (DEE), the risk is already substantially elevated in childhood, with up to 25% of SUDEP cases occurring before the age of 20 years [15, 63], highlighting the vulnerability of this population. Consequently, SUDEP represents the second most common neurological cause of years of potential life lost, surpassed only by ischemic stroke [80].

SUDEP is defined as a sudden, unexpected, non‑traumatic and non‑drowning death in a person with epilepsy, occurring from a previously stable state of health, which may or may not be preceded by an epileptic seizure [48]. The event may be witnessed or unwitnessed; death due to status epilepticus must be excluded, and a post‑mortem examination is required to establish the diagnosis. In the absence of an autopsy, the term ‘probable SUDEP’ is used. Because status epilepticus (SE) as a cause of death precludes a diagnosis of SUDEP, a potential SE should be specifically ruled out—particularly in individuals with a prior history of SE [4] or in otherwise vulnerable patient groups [78]. Thus, the diagnosis of SUDEP remains a diagnosis of exclusion. A further subcategory, SUDEP Plus, is used when coexisting medical conditions—such as cardiac arrhythmias—may have contributed to the fatal event but are insufficient to fully account for death on their own.

Because a definitive diagnosis of SUDEP requires an autopsy, this may hinder the ascertainment of all cases and thereby affect the quality of registry‑based studies [50]. In particular, in complex cases, in older individuals, and in the presence of multiple comorbidities, determining the exact cause of death becomes increasingly challenging [34].

Despite decades of research, the underlying mechanisms of SUDEP remain incompletely understood. Recent large‑scale prospective multicenter studies have substantially advanced our understanding of SUDEP [49, 61]. In this review, the findings of these studies are integrated and contextualized within the existing body of evidence. This narrative review synthesizes the current state of research on SUDEP, with a particular focus on clinical data and recent advances in pathophysiology and delineates prospective directions for future investigations and prevention.

## Epidemiology

Several studies of varying methodological quality have examined the incidence of SUDEP in past years. Table 1 summarizes the findings of major recent studies as well as those of a large meta‑analysis outlining study results performed until then. Across all PWE, the SUDEP risk is approximately 1.1–1.6 per 1,000 patient‑years (py) [29, 35, 74]. In cohorts with treatment‑refractory epilepsy, the risk is around 2.6–4.8 per 1,000 py [15, 49, 61]. The highest risk is estimated for individuals with Dravet syndrome, at nearly 10 per 1,000 py [10, 46], with vulnerability to SUDEP being concentrated in the first two decades of life. Depending on the age at onset of epilepsy and the epilepsy syndrome, the cumulative lifetime risk of SUDEP is estimated to be between 5% and 20% [10, 66, 80]. There appears to be a declining trend in SUDEP incidence in recent years, even in the absence of specific interventions, although the underlying cause remains unclear [9, 82].

**Table 1:** Selected studies from recent years on the incidence of SUDEP

Incidence / 1000 py (CI)	Patient cohort	Method	Study
9.32 (4.46–19.45)	Patients with Dravet syndrome	Prospective, cohort	Cooper 2016
4.76 (3.37–6.53)	Children and adults, evaluated in epilepsy monitoring units	Prospective, cohort	Ochoa-Urrea 2025
2.8 (1.6–4.3)	Patients with genetic DEE	Retrospective, cohort	Donnan 2023
2.64 (1.36–3.92)	Presurgical, > 15 years of age	Prospective, cohort	Ryvlin 2026
1.65	PWE aged 1–49 years	Retrospective, population-based cohort	Klovgaard 2021
1.20 (0.64–2.32)	Adults with epilepsy	Metaanalysis	Harden 2017
1.20	Population-based	Retrospective, national registry	Sveinsson 2017
1.11 (0.63–1.79)	Children with epilepsy	Retrospective	Keller 2018
0.8	Children with epilepsy	Retrospective, cohort	Gronborg 2014
0.22 (0.16–0.31)	Children with epilepsy	Metaanalysis	Harden 2017

CI: confidence interval, py: patient years

Findings regarding SUDEP rates in children are heterogeneous. While some studies have demonstrated a markedly lower risk in pediatric populations [29], other analyses have found no significant differences [26, 33].

## Risk Factors and Biomarker

A broad range of clinical, demographic, and situational factors have been identified that modify SUDEP risk. Most reported SUDEP cases are observed in individuals of middle age (19–49 years), and nearly two thirds of all SUDEP cases occur while the individual is lying in bed, mostly in prone position [27, 75]. The most frequently investigated risk factors are summarized in Table 2. Drawing on the most salient risk factors, risk stratification scores have been developed to facilitate the identification of PWE at particularly elevated risk of SUDEP [79].

The most consistently replicated and quantitatively strongest risk factor for SUDEP is the presence and frequency of BTCS, especially when nocturnal, and the risk further increases as a function of BTCS frequency [13]. Risk increases measurably from 1 to 2 BTCS/year [30], with a steep rise at ≥ 3 BTCS/year, and the highest odds ratios observed at ≥ 4 BTCS/year [76]. The risk associated with nocturnal seizures likely reflects seizure occurrence during sleep rather than the time of day per se. Seizures during an afternoon nap may therefore also elevate SUDEP risk; because the cited studies predominantly examined “nocturnal seizures” rather than “seizures occurring during sleep,” we retain the former term here. In PWE with frequent or nocturnal BTCS, the SUDEP risk is highest during the first 5 years after the epilepsy diagnosis and thereafter seems to decline with increasing epilepsy duration [77]. The risk also appears to be increased in individuals with childhood‑onset epilepsy and a long disease duration [30]. Elevated risk in the early post‑diagnostic period is confined to patients with frequent or nocturnal BTCS. In contrast, the association with prolonged disease duration likely reflects cumulative exposure to uncontrolled seizures; these patterns therefore represent distinct epidemiological phenomena in different patient subgroups rather than a contradiction. However, the effect of age at epilepsy onset could not be consistently replicated across studies, and no significant associations were observed between SUDEP and the number of antiseizure medications (ASM), or history of depression [61].

The strongest social risk factor for SUDEP is the individual’s living circumstances: PWE who live alone exhibit a six-fold higher SUDEP-risk compared with those who share a bedroom [76]. Male sex exhibits modest effect size and inconsistent statistical significance across studies, which did not reach statistical significance in all analyses.

The combination of multiple risk factors further amplifies the risk of SUDEP. PWE who are non‑adherent to their prescribed ASM, live alone, and experience nocturnal BTCS exhibit a more than 350‑fold higher SUDEP risk compared with PWE who are free of BTCS and share a bedroom [83].

**Table 2:** SUDEP Risk Factors

Risk factor	OR	Study
Sex (male vs. female)	12.61 (1.49-106.83)	Ryvlin 2026
	1.68 (0.9–3.13)**	Ochoa-Urrea 2025
	1.42 (1.07–1.88)	Hesdorffer 2011
BTCS ≥ 1/year	*5.64 (0.89–35.9)*	Ryvlin 2026
History of BTCS (yes vs. no)	10.56 (3.86–28.86)	Sveinsson 2020
1–3 BTCS during preceding year (vs. no)	19.51 (11.94–31.88)	Sveinsson 2020
4–10 BTCS during preceding year (vs. no)	28.24 (15.36–51.92)	Sveinsson 2020
> 10 BTCS during preceding year (vs. no)	26.38 (14.62–47.61)	Sveinsson 2020
1–2 BTCS/year	5.07 (2.94–8.76)	Hesdorffer 2011
≥ 3 BTCS/year	15.46 (9.92–24.10)	Hesdorffer 2011
	3.10 (1.64–5.87)**	Ochoa-Urrea 2025
Predominantly nocturnal seizure (yes vs. no)	5.95 (1.23–28.74)	Ryvlin 2026
History of nocturnal BTCS (vs. no nocturnal seizures)	8.44 (5.91–12.04)	Sveinsson 2020
Nocturnal BTCS during preceding year	12.98 (8.61–19.56)	Sveinsson 2020
Focal epilepsy (vs. generalized)	1.48 (1.0-2.2)	Sveinsson 2020
Onset Age < 16 years	1.72 (1.23–2.40)	Hesdorffer 2011
Age at epilepsy onset	*0.96 (0.86–1.07)*	Ryvlin 2026
Duration of epilepsy > 15 (vs. ≤15) years	1.95 (1.45–2.63)	Hesdorffer 2011
ASM polytherapy (vs. monotherapy)	1.53 (0.73–3.21)**	Ochoa-Urrea 2025
	*1.67 (0.98–2.84)*	Sveinsson 2020
	1.95 (1.09–3.47)	Hesdorffer 2011
BMI > 30 (yes vs. no)	25.97 (1.99-339.56)	Ryvlin 2026
Substance abuse	2.57 (1.63–4.05)	Sveinsson 2020
Intellectual disability	2.48 (1.79–3.42) *	Sveinsson 2020
	1.41 (0.68–2.89)**	Ochoa-Urrea 2025
Cardiac comorbidities	2.34 (0.98–5.59)**	Ochoa-Urrea 2025
Mycarditis, cardiomyopathy, arrhythmias	*1.19 (0.71-2.00)*	Sveinsson 2020
Ischemic heart disease	*0.65 (0.35–1.20)*	Sveinsson 2020
Living alone (vs. sharing bedroom)	6.11 (4.04–9.22)	Sveinsson 2020
Sharing household, but not bedroom (vs. sharing bedroom)	2.43 (1.36–4.32)	Sveinsson 2020
Living alone (vs. living with someone)	7.62 (3.94–14.71)**	Ochoa-Urrea 2025

*not significant after adjusting for generalized tonic-conic seizure frequency

** Hazard Ratio

Italic: statistically not significant. BTCS: bilateral tonic-clonic seizure, ASM: antiseizure medication, BMI: Body mass index

Focal epilepsies carry a higher risk of SUDEP than genetic generalized epilepsies; however, when BTCS persist and the frequency exceeds four BTCS per year, the difference between focal and generalized epilepsies is no longer observed [77]. Accordingly, the elevated SUDEP risk associated with focal epilepsies is best interpreted as reflecting their, on average, less effective seizure control.

Compared with controls, PWE who died of SUDEP were significantly more likely to have a body‑mass index ≥ 30, male sex, and predominantly nocturnal seizures [61].

In contrast to adult SUDEP cases, nearly half of pediatric cases have been reported to involve a preceding infection [87]. Children with global developmental delay are more frequently affected by SUDEP; however, SUDEP can rarely also occur in the context of normal neurodevelopment and at any age and any epilepsy syndrome during childhood. Even in cases of “benign” epilepsy with centrotemporal spikes, SUDEP cases have been reported [45].

SUDEP remains a stochastic event: the identified risk factors increase the likelihood of SUDEP, yet a residual risk must always be assumed for every person with epilepsy, even in absence of those factors.

### Biomarkers

Various ictal and interictal electroclinical and autonomic characteristics are discussed as biomarkers for SUDEP risk. Biomarkers currently under the most intensive investigation include EEG characteristics, respiratory and sleep parameters, cardiac parameters, and genetic markers. Identifying biomarkers for SUDEP may help to detect PWE at high-risk for SUDEP, and may additionally advance our understanding of the underlying pathophysiology. Ictal markers could facilitate potential life‑saving interventions, such as nocturnal monitoring with caregiver notification or on‑demand stimulation to restore arousal during the postictal period. However, to date, no biomarkers have been validated that reliably discriminate between seizures that confer a high risk of SUDEP and those that do not. Candidate biomarkers that have been discussed in recent years are summarized in Table 3. Two prospective, multicenter studies using long‑term video‑EEG monitoring (VEM) and data on heart- and respiratory rates have recently been published with the aim of validating electroclinical biomarkers of SUDEP [49, 61]. In the methodologically rigorous, carefully designed, multicenter prospective studies with several years of follow‑up, 56 SUDEP cases in total were identified among more than 3,500 enrolled PWE. This represents an impressive number relative to the SUDEP incidence rate, yet it still poses challenges for statistical analyses.

Prolonged postictal generalized EEG suppression (PGES) has widely been discussed as a potential SUDEP biomarker [73]; however, the existing evidence remains heterogeneous. Prolonged postictal generalized EEG suppression (PGES) has been widely discussed as a potential SUDEP biomarker [73]; however, the existing evidence remains heterogeneous. A central hypothesis in the pathophysiology of SUDEP involves impaired arousal following a BTCS, with propagation of the generalized cortical suppression of brain activity to subcortical structures. In a retrospective case–control study of ten adult patients who later died of SUDEP, a PGES duration exceeding 50 s was significantly associated with subsequent SUDEP [38], others failed to demonstrate a significant effect of PGES on SUDEP risk [32]. In the study by Ochea‑Urrea et al., (2025), postictal EEG suppression exhibited a minor effect on subsequent SUDEP risk. Due to the low number of recorded BTCS in the study cohort, the correlation between PGES and SUDEP risk could not be further evaluated statistically by Ryvlin and colleagues. The current body of evidence indicates that the extent and duration of PGES alone is not sufficient to reliably distinguish between patients at risk of SUDEP and those not at risk.

**Table 3:** Possible biomarker of SUDEP

Biomarkers	Metrics	Study
**Respiratory Markers**		
Postictal central apnoea, per 10s increment, HR	1.32 (1.14–1.54)	Ochoa-Urrea 2025
Ictal central apnoea duration, per 10s increment, HR	1.11 (1.05–1.18) ‡	Ochoa-Urrea 2025
Total hypoxaemia duration, per 10s increment, HR	*1.03 (0.95–1.12)*	Ochoa-Urrea 2025
**Sleep related breathing disorders**		
SA-SDQ > = 26 in female or > = 29 in male, OR	*2.48 (0.13–47.8)*	Ryvlin 2026
Coefficient of inter-breath interval variability, SUDEP vs. low-SUDEP risk Group, SD	0.15 (0.09) vs. 0.08 (0.03)	Magana-Tellez 2025
**Cardial Markers**		
Any arrhythmia, HR	4.96 (1.74–14.2)	Ochoa-Urrea 2025
Heart Rate Variabilitiy, OR	*0.99 (0.92–1.08)*	Ryvlin 2026
Interictal short-term heart rate variability, low- and high frequency power, HR	*0.99–1.01 (0.98–1.02)*	Ochoa-Urrea 2025
**EEG Markers**		
Postictal generalised EEG suppression duration, per 10s increment, HR	1.12 (1.03–1.22) ‡	Ochoa-Urrea 2025
Epileptogenic zone (extratemporal vs. temporal), OR	37.84 (3.21-446.15)	Ryvlin 2026
Sleep Slow Wave Activity power, slope (standardized error of the mean) (SUDEP vs. low SUDEP risk patients)	0.005 (0.003)vs. -0.003 (0.002)	Magana-Tellez 2025
**Genetic Markers**		
KCNH2 LOF variants, OR	2.7 (1.1–7.4)	Soh 2021
SCN5A, KIF6, TBX	n.a.	Ge 2020
SCN5A	n.a.	Soh 2022
KCNA1, SCN1A, SCN2A, SCN8A, DEPDC5, KCNQ1, KCNH2, SCN5A	n.a.	Bagnall 2017

‡ after excluding near-SUDEP and possible SUDEP-cases, findings were no longer statistically significant

SA-SDQ: Sleep Apnea Scale of the Sleep Disorders Questionnaire

HR Hazard Ratio, OR Odds Ratio, SD Standard Deviation

Both (focal to) BTCS and seizures remaining focal may exhibit interictal and postictal apneas with resulting hypoxemia. The respiratory abnormalities and the severity of the hypoxemia have likewise been discussed as a potential SUDEP biomarker. The incidence and proportion of peri‑ictal oxygen desaturation were identical in SUDEP and non-SUDEP groups [61]. However, other interictal respiratory markers appear to be more useful as potential biomarkers.

A mathematical model successfully discriminated between SUDEP and non‑SUDEP cases by extracting respiratory signals from ictal electrocardiogram [24]. In SUDEP cases, a significantly increased ictal cardiorespiratory coupling strength has been found, leading to a shift in cardiorespiratory dynamics to higher frequencies. The interbreath interval is a metric of respiratory variability, and high variability reflects reduced central respiratory control. In PWE and a high seizure burden, increases in the variability of the interbreath interval have been demonstrated interictally in Non-REM sleep [62]. This has been observed particularly in PWE who were classified as being at high risk for SUDEP or who subsequently died from SUDEP [42]. A potential explanation lies in the presumed habituation of central chemoreceptors to rises in CO₂ due to frequent apneas with associated hypoxemia and hypercapnia caused by recurrent epileptic seizures. This progressive central cardiorespiratory downregulation has been demonstrated in a SUDEP mouse model [31].

Most SUDEP cases occur during sleep following a BTCS. However, the underlying reason for this association remains the subject of ongoing research. BTCS arising from sleep do not differ from wake-related BTCS with respect to the extent of postictal immobility [51]. Several studies have demonstrated prolonged PGES following seizures arising from sleep [37, 51]. While some studies did not report longer hypoxic episodes [51], others found that the depth of desaturation was more pronounced in sleep-related seizures [37]. Although the severity of postictal hypoxemia and PGES does not correlate directly with SUDEP risk, their increased occurrence following sleep‑related seizures may indicate a heightened vulnerability of the system.

SUDEP patients show a disrupted sleep architecture—characterized by abnormal proportions of deep sleep—independent of epileptic seizures [42]. This study, which analyzed additional data from the [49] cohort to examine the relationship between SUDEP, sleep, and respiration, was the first to demonstrate a clear association between disrupted sleep homeostasis and SUDEP. Patients who later died from SUDEP exhibited an unphysiological lack of decline in slow‑wave sleep across the night. In patients with Dravet syndrome, an impairment of slow‑wave sleep attributable to seizures and interictal discharges has been demonstrated [11] and may partly account for the syndrome’s markedly elevated SUDEP risk. Slow wave sleep is considered crucial for consolidation of newly acquired information in memory and neural plasticity, via downscaling synaptic strength to a baseline level that is energetically sustainable [84].

Sleep disorders are common in PWE [89], and early recognition and treatment can improve seizure frequency and might potentially reduce SUDEP risk [56]. PWE have a higher prevalence of obstructive sleep apnea (OSA) than the general population (Odds Ratio 2.36) [40], independent of seizure burden and the type of ASM used. Given that individuals with OSA face an elevated risk of nocturnal sudden cardiac death, this prompted the question of whether comorbid OSA may similarly contribute to an increased risk of SUDEP in PWE. To date, neither the questionnaire-based OSA screening approach employed by Ryvlin et al., [61], nor the use of established SUDEP risk scores [52] provide evidence for an association.

In addition, cardiac biomarkers are a frequent focus of investigation. The occurrence of ‘any arrhythmia’ during VEM has been reported to exert an effect on SUDEP risk [49]. Although heart rate variability has been suggested as a potential SUDEP biomarker [68], neither of the two prospective cohorts was able to validate this association [49, 61].

Several risk genes have been associated with SUDEP. These encompass genes associated with severe epilepsies, including DEEs; channelopathies expressed in both the brain and the heart; and, not least, genetic variants known to underlie long‑QT syndrome [1]. Because SUDEP cases show higher frequencies of genes predisposing to long‑QT syndrome, this supports a cardiac origin of the SUDEP cascade in at least a subset of patients.

In children, several associations between seizure characteristics and cardiopulmonary disturbances during seizures have been described. Overall, most prominent ictal distress with decrease of respiration or heart rate (apnea, bradypnea, bradycardia, and desaturation) was seen in younger boys with focal to BTCS, or left temporal and longer-duration seizures, who were taking multiple ASM [67]. However, these studies were unable to demonstrate a direct association of those markers with pediatric SUDEP cases.

## Pathophysiological considerations

In PWE who died from SUDEP during inpatient VEM with simultaneous cardiorespiratory recordings, a uniform cascade preceding SUDEP was observed: following a focal‑to‑BTCS, an inconsistent brief phase of rapid breathing occurred, followed by central apnea, which subsequently progressed to bradycardia and ultimately terminal asystole [59]. These data support the hypothesis that most SUDEP cases represent a primarily respiratory rather than primarily cardiac event.

**Fig. 1:**
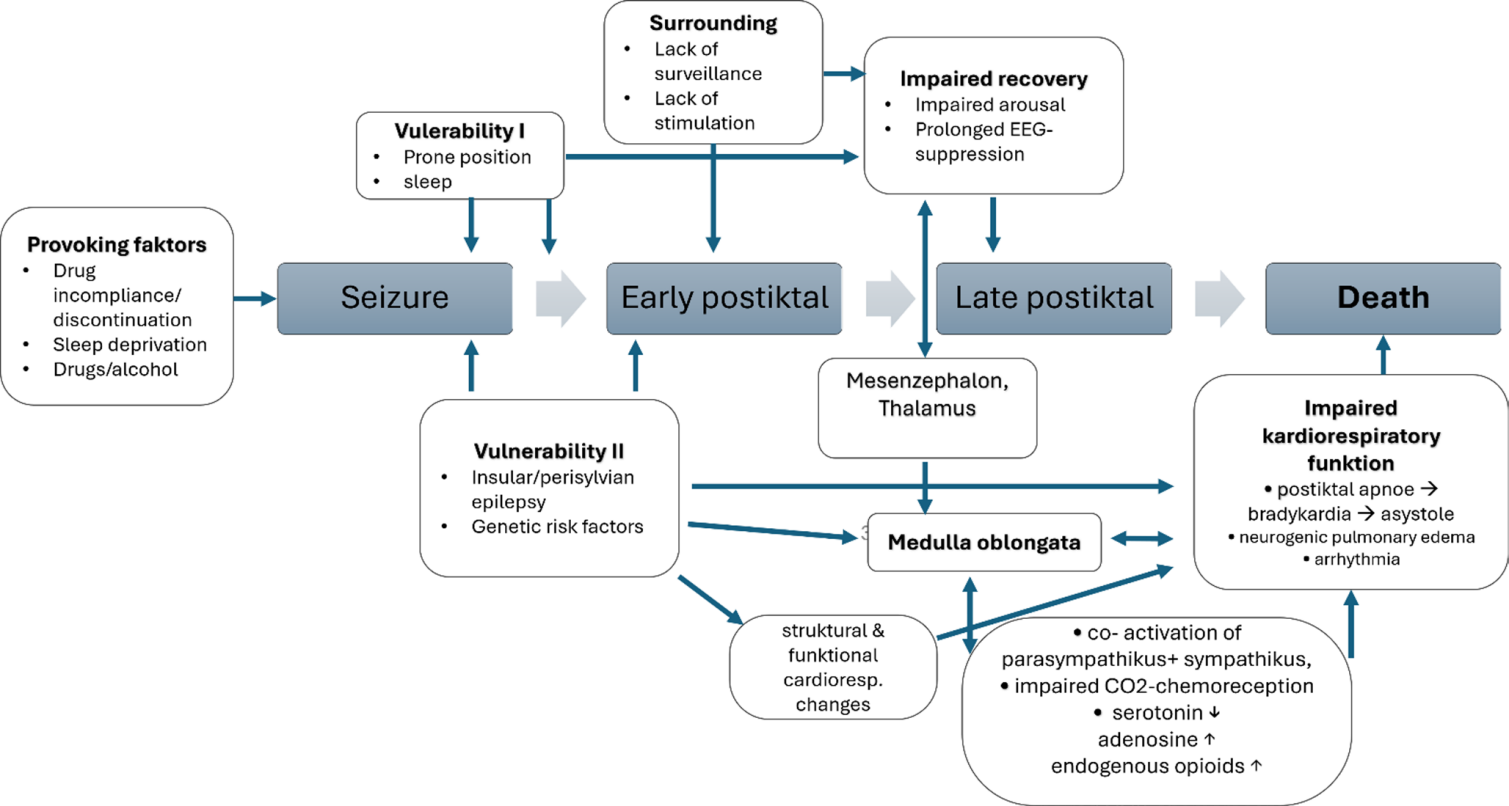
Pathophysiological model of SUDEP. Modified from [14]

Based on established risk factors and the observed SUDEP events described above, the current conceptual model of the SUDEP cascade is as follows: in most cases, a BTCS precedes the event. This is followed by a pathological impairment of arousal, which may be facilitated by seizures arising from sleep and by insufficient external stimulation (Fig. 1). The pathologic impaired arousal response and postictal dysregulation lead to adenosine‑mediated inhibition of serotonergic neurons, resulting in respiratory depression. Primary respiratory arrest subsequently triggers bradycardia and ultimately asystole [14]. Less commonly, SUDEP may arise from seizures that remain focal, and SUDEP cases in refractory PWE without an immediately preceding epileptic seizure have also been reported [39].

The involvement of the brainstem became evident through a significant reduction in brainstem volume in SUDEP cases, especially in the ventrolateral medulla, including the pre-Bötzinger complex (PBC). The PBC refers to a cluster of neurons closely associated with inspiratory neuronal activity [19]. It is part of the ventral respiratory group in the medulla oblongata and plays a critical role in generating the respiratory rhythm [69]. Raphe serotonergic neurons are proposed to play a role in mediating CO_2_ chemosensitivity, enhancing the respiratory drive by detecting acidosis resulting from increases in CO₂ [2]. In addition, the PBC is widely and paucisynaptically connected to higher brain centers that regulate arousal and excitability more generally, such that respiratory brain function is intimately connected with many other rhythmic and cognitive functions of the brain and central nervous system. Severe hypoxia and hypercapnea caused by increasingly long and frequent apneas, as in the context of BTCS, can itself cause neuronal death [25]. The increased interbreath interval variability, which—as described above—may serve as a biomarker of SUDEP, may potentially be attributable to these post-hypoxic alterations. The contribution of endogenous opioids to postictal apnea and immobility has been discussed; however, administration of the opioid antagonist naloxone has not been shown to affect postictal hypoxemia, but might reduce the duration of post-ictal immobility [55].

In addition to the brainstem, supratentorial centers are also involved in the regulation of respiration. For example, stimulation of the amygdala and seizures involving it trigger apnea via direct connection to the PBC and pontine centers. Accordingly, peri‑ictal central apneas occur not only after BTCS but also in approximately 6% of seizures that remain focal, associated with an enlarged amygdala [88], more pronounced ipsilateral to the epileptogenic zone [47].

In contrast to respiratory function, cardiac activity is inherently more autonomous, though subject to modulation by central neural influences. Consistent effects on heart rate and heart rate variability were observed in the anterior cingulate, amygdala, insula, and prefrontal cortex, among others [57]. Thus, electrical stimulation of the insula can elicit either increases or decreases in heart rate, depending on the specific region stimulated [81]. In the SUDEP group, extratemporal epileptogenic foci were observed more frequently compared with the controls, largely driven by a higher proportion of perisylvian foci and, to a lesser extent, frontal foci in SUDEP cases, whereas posterior epileptogenic zones were absent among those who died [61]. Therefore, in focal epilepsies, the exact location of the epileptogenic zone may influence SUDEP risk, given the distinct autonomic network connections associated with different cortical and subcortical regions.

Although respiratory dysfunction is currently central to pathophysiological considerations, primarily cardio-arhythmic events may contribute or even predominate in other cases. Although the majority of documented SUDEP cases appear to follow the canonical apnea–bradycardia–asystole cascade, several reports describe various cardiac arrhythmias in a subset of cases [17]. Approximately 14% of BTCS are accompanied by potentially SUDEP‑associated cardiac arrhythmias, with bradycardic disturbances in particular showing a small but significant association with subsequent SUDEP [85].

### Insights from animal models

Heterogeneity among SUDEP cases with respect to underlying mechanisms and contextual circumstances complicate efforts to model and predict these events. Rodent models have proven valuable for investigating mechanisms underlying seizure‑induced physiological dysfunction. However, since SUDEP risk is artificially increased in these models through specific induction (genetic or chemical/electrical), and the etiologies are not comparable to the human spectrum the translatability of results remains uncertain. Accordingly, the following section highlights only exemplary findings from animal studies.

Animal models suggest that prolonged postictal apnoea is mediated by adenosine, whereas serotonin exerts a respiratory‑stimulating effect. Therefore, serotonergic or direct activation of the periaqueductal gray may be a useful approach for SUDEP prevention [18], and use of fenfluramine in Dravet patients pointed towards a reduced risk of SUDEP in this cohort [12].

To elucidate the contribution of adenosine to peri‑ictal dysregulation, rats in a SUDEP model received high doses of nonselective adenosine receptor antagonists (caffeine and theophylline). If a seizure‑induced rise in adenosine exerts an inhibitory effect on respiration, this effect should be at least partially reversed by adenosine antagonists. Unexpectedly, the treated animals showed an increased risk of seizure-related deaths [54].

Audiogenically induced seizures in mice produced a pattern of physiological decompensation that normalized in some animals but resulted in death in others, partly with a marked delay after the seizures. This demonstrates that SUDEP-related cascades may also occur with a temporal delay following epileptic events [16].

## Prevention of SUDEP

Pathophysiological considerations form the basis of current approaches aimed at reducing SUDEP risk, although the overall level of evidence remains low. The strongest evidence to date relates to nocturnal supervision with a supervising individual sharing the same bedroom, or to special precautions such as a listening device [36]. Given that the BTCS occurrence is the most robustly established SUDEP risk factor, optimal seizure control is essential. The addition of newer, highly effective ASMs—including cenobamate and, in the case of Dravet syndrome, fenfluramine—has been linked to reduced SUDEP rates in the examined populations [12, 71]. In patients with Dravet syndrome, fenfluramine may reduce SUDEP risk via improved seizure control, favorable effects on sleep architecture, and its serotonergic mechanism of action [11, 12]. Meta‑analyses of randomized, placebo‑controlled adjunctive ASM trials in treatment‑refractory epilepsy have reported a SUDEP incidence in placebo arms more than seven times higher than in active‑treatment arms [58]. This finding underscores the need to optimize pharmacotherapy even in refractory patients to reduce SUDEP risk and raises ethical concerns about trial designs that expose participants to suboptimal treatment; alternative designs that minimize or eliminate placebo exposure merit consideration.

Epilepsy surgery for refractory focal epilepsy not only achieves superior seizure control compared with further pharmacotherapy but also reduces mortality, notably by lowering SUDEP rates [21, 65]. The effect of neurostimulation devices on SUDEP rates has been studied primarily in relation to vagus nerve stimulators, but remains inconclusive: While a large cohort study documented a significant long‑term decrease in SUDEP risk among patients with refractory epilepsy receiving VNS therapy [60], other investigations failed to detect a similar effect [23].

Early and explicit counseling of patients and families is crucial, emphasizing that treatment adherence and good sleep hygiene may contribute to risk reduction. Current studies show limited overall knowledge on SUDEP among patients and health care providers, the latter not informing all patients about SUDEP [64, 72]. The central objective of counseling is to optimize risk reduction while preserving the patient’s maximal independence [8]. PWE welcome early counselling about SUDEP, and such information does not increase depressive symptoms or anxiety [86].

The patient’s position prior to seizure onset predicts their postictal position [44]. Patients are advised to sleep supine and to consider firm pillows; however, the level of evidence supporting these recommendations is low [43]. We routinely discuss options for nocturnal monitoring with patients and families and offer first‑aid training to caregivers. Interventions by caregivers during and after an epileptic seizure, as well as regular training in basic cardiopulmonary resuscitation may contribute to a reduced SUDEP risk, particularly in high‑risk populations [41, 46]. Tactile or verbal stimulation during the postictal period may help restore arousal and breathing, thereby preventing SUDEP and reducing the need for resuscitation measures. Various seizure‑detection devices are available for nighttime supervision. Automated seizure‑detection systems primarily analyze motor manifestations of seizures—most commonly via surface electromyography and accelerometry—and seizure‑related autonomic changes, with electroencephalographic signals contributing to a lesser extent [5]. These systems attain high sensitivity and low false‑alarm rates for (focal to or generalized) BTCS. Detection of focal seizures that lack prominent motor or autonomic signatures, however, remains suboptimal. Automated seizure‑detection devices that immediately notify caregivers of BTCS could potentially reduce SUDEP, although empirical evidence for this effect is currently lacking [6, 7].

Other approaches to SUDEP prevention require further research and development before they can be put into practice. These include new methods of seizure detection (warning before rather than after a seizure), drug-based approaches, and technical devices. Ideally, detection systems would provide pre‑ictal rather than post‑ictal alerts, and ongoing research is exploring seizure‑forecasting approaches, but remain still experimental and not available for clinical use [3].

In addition, future preventive strategies are likely to rely on precision‑medicine concepts targeting individual, for example genetic, risk profiles. Emerging evidence on serotonergic pharmacological interventions also shows initial promise [70]. The use of antisense oligonucleotides (ASO) in Dravet syndrome has already been shown to drastically reduce SUDEP rates in animal models [28], and first studies in humans are imminent. A clinical trial investigating the effect of an opioid antagonist on the duration of postictal immobility is under way.

Other promising approaches to SUDEP prevention are even further from clinical translation. In animal models, postictal apnea has been shortened through the use of diaphragmatic pacing, which in turn has been shown to reduce SUDEP rates in a rodent model [53]. Such invasive interventions, however, are applicable in humans only if suitable biomarkers can be identified that allow the detection of PWE at the highest risk of SUDEP.

## Conclusion

Although PWE — particularly those with drug‑resistant courses — face a substantial lifetime risk of SUDEP, the low annual incidence remains a major challenge for research. Especially due to the hypothesis that, alongside the “typical” SUDEP cascade—occurring during sleep, in the prone position, after BTCS with postictal apnea progressing to asystole—there may also be “atypical” SUDEP pathways, investigating these phenomena will require sustained scientific effort and very large, multicenter collaborations and datasets.

Prevention strategies based on the evidence reported include informing patients and caregivers, improvement of compliance, and avoidance of BTCS. The strongest empirical support concerns nocturnal supervision and rigorous control of BTCS, the principal risk factor. Early counselling of patients and families promotes adherence, while practical measures—supine sleeping, and postictal tactile or verbal stimulation—may mitigate risk. Automated seizure‑detection devices show high sensitivity for BTCS but perform poorly for nonmotor focal seizures, and direct evidence that such devices reduce SUDEP is lacking. Promising research avenues include pre‑ictal forecasting, serotonergic interventions, and precision‑medicine approaches.

## Data Availability

Data sharing is not applicable to this article as no datasets were generated or analyzed during the current study.

## Abbreviations

ASM: Antiseizure-Medication
ASO: Antisence Oligonucleotide
BTCS: Bilateral tonic clonic seizure
DEE: Developmental and epileptic encephalopathies
PBC: Pre-Bötzinger Complex
PGES: Postictal generalized EEG Suppression
PWE: People with epilepsy
SUDEP: Sudden unexpected death in epilepsy patients

## References

[1] Bagnall, R. D., Crompton, D. E., & Semsarian, C. (2017). Genetic Basis of Sudden Unexpected Death in Epilepsy. *Frontiers In Neurology*, *8*, 348. 10.3389/fneur.2017.00348 28775708 10.3389/fneur.2017.00348 PMC5517398

[2] Ballantyne, D., & Scheid, P. (2000). Mammalian brainstem chemosensitive neurones: linking them to respiration in vitro. *J Physiol 525 Pt*, *3*(Pt 3), 567–577. 10.1111/j.1469-7793.2000.00567.x 10.1111/j.1469-7793.2000.00567.x PMC2269968 10856112

[3] Baud, M. O., Proix, T., Gregg, N. M., Brinkmann, B. H., Nurse, E. S., Cook, M. J., & Karoly, P. J. (2023). Seizure forecasting: Bifurcations in the long and winding road. *Epilepsia 64 Suppl*, *4*(Suppl 4), S78–S98. 10.1111/epi.17311 10.1111/epi.17311 PMC9681938 35604546

[4] Bauer, K., Rosenow, F., Knake, S., Willems, L. M., Kamppi, L., & Strzelczyk, A. (2023). Clinical characteristics and outcomes of patients with recurrent status epilepticus episodes. *Neurol Res Pract*, *5*(1), 34. 10.1186/s42466-023-00261-9 37438822 10.1186/s42466-023-00261-9 PMC10339656

[5] Baumgartner, C., Baumgartner, J., Lang, C., Lisy, T., & Koren, J. P. (2025). Seizure Detection Devices. *J Clin Med*, *14*(3). 10.3390/jcm14030863 10.3390/jcm14030863 PMC11818620 39941534

[6] Beniczky, S., Wiebe, S., Jeppesen, J., Tatum, W. O., Brazdil, M., Wang, Y., Herman, S. T., & Ryvlin, P. (2021). Automated seizure detection using wearable devices: A clinical practice guideline of the International League Against Epilepsy and the International Federation of Clinical Neurophysiology. *Clinical Neurophysiology*, *132*(5), 1173–1184. 10.1016/j.clinph.2020.12.009 33678577 10.1016/j.clinph.2020.12.009

[7] Bernini, A., Dan, J., & Ryvlin, P. (2024). Ambulatory seizure detection. *Current Opinion In Neurology*, *37*(2), 99–104. 10.1097/WCO.0000000000001248 38328946 10.1097/WCO.0000000000001248

[8] Bertinat, A., Kerr, M., Cramer, J. A., & Braga, P. (2020). Living safely with epilepsy: a key learning review. *Epileptic Disorders : International Epilepsy Journal With Videotape*, *22*(4), 364–380. 10.1684/epd.2020.1190 32763871 10.1684/epd.2020.1190

[9] Cihan, E., Devinsky, O., Hesdorffer, D. C., Brandsoy, M., Li, L., Fowler, D. R., Graham, J. K., Karlovich, M. W., Yang, J. E., Keller, A. E., Donner, E. J., & Friedman, D. (2020). Temporal trends and autopsy findings of SUDEP based on medico-legal investigations in the United States. *Neurology*, *95*(7), e867–e877. 10.1212/WNL.0000000000009996 32636323 10.1212/WNL.0000000000009996 PMC7605498

[10] Cooper, M. S., McIntosh, A., Crompton, D. E., McMahon, J. M., Schneider, A., Farrell, K., Ganesan, V., Gill, D., Kivity, S., Lerman-Sagie, T., McLellan, A., Pelekanos, J., Ramesh, V., Sadleir, L., Wirrell, E., & Scheffer, I. E. (2016). Mortality in Dravet syndrome. *Epilepsy Research*, *128*, 43–47. 10.1016/j.eplepsyres.2016.10.006 27810515 10.1016/j.eplepsyres.2016.10.006

[11] Cossu, A., Proietti, J., Ghobert, L., Rinaldi, L., Dalla Bernardina, B., Darra, F., & Cantalupo, G. (2025). Seizures influence sleep macrostructure and the sleep-wake circadian rhythm in Dravet syndrome. *Epilepsia*, *66*(9), 3269–3281. 10.1111/epi.18451 40347411 10.1111/epi.18451

[12] Cross, J. H., Galer, B. S., Gil-Nagel, A., Devinsky, O., Ceulemans, B., Lagae, L., Schoonjans, A. S., Donner, E., Wirrell, E., Kothare, S., Agarwal, A., Lock, M., & Gammaitoni, A. R. (2021). Impact of fenfluramine on the expected SUDEP mortality rates in patients with Dravet syndrome. *Seizure*, *93*, 154–159. 10.1016/j.seizure.2021.10.024 34768178 10.1016/j.seizure.2021.10.024

[13] DeGiorgio, C. M., Markovic, D., Mazumder, R., & Moseley, B. D. (2017). Ranking the Leading Risk Factors for Sudden Unexpected Death in Epilepsy. *Frontiers In Neurology*, *8*, 473. 10.3389/fneur.2017.00473 28983274 10.3389/fneur.2017.00473 PMC5613169

[14] Devinsky, O., Hesdorffer, D. C., Thurman, D. J., Lhatoo, S., & Richerson, G. (2016). Sudden unexpected death in epilepsy: epidemiology, mechanisms, and prevention. *Lancet Neurology*, *15*(10), 1075–1088. 10.1016/S1474-4422(16)30158-2 27571159 10.1016/S1474-4422(16)30158-2

[15] Donnan, A. M., Schneider, A. L., Russ-Hall, S., Churilov, L., & Scheffer, I. E. (2023). Rates of Status Epilepticus and Sudden Unexplained Death in Epilepsy in People With Genetic Developmental and Epileptic Encephalopathies. *Neurology*, *100*(16), e1712–e1722. 10.1212/WNL.0000000000207080 36750385 10.1212/WNL.0000000000207080 PMC10115508

[16] Eilbes, M., Gallo, A., Osmani, W. A., Bittencourt-Silva, P., Manis, A. D., & Hodges, M. R. (2025). Unique features of seizure-induced cardiorespiratory failure in SS(Kcnj16-/-) rats: Implications for sudden unexpected death in epilepsy. *Epilepsia*, *66*(7), 2578–2591. 10.1111/epi.18360 40067249 10.1111/epi.18360 PMC12291026

[17] Ermongkonchai, T., Kwok, M., Prinsloo, D., Hakak-Zargar, B., Nurse, E., Cook, M. J., & Ha, F. J. (2025). Cardiac arrhythmias in sudden unexpected death in epilepsy: A systematic review. *Epilepsy & Behavior*, *171*, 110582. 10.1016/j.yebeh.2025.110582 40618461 10.1016/j.yebeh.2025.110582

[18] Faingold, C. L., & Feng, H. J. (2023). A unified hypothesis of SUDEP: Seizure-induced respiratory depression induced by adenosine may lead to SUDEP but can be prevented by autoresuscitation and other restorative respiratory response mechanisms mediated by the action of serotonin on the periaqueductal gray. *Epilepsia*, *64*(4), 779–796. 10.1111/epi.17521 36715572 10.1111/epi.17521 PMC10673689

[19] Feldman, J. L., Negro, D., C. A., & Gray, P. A. (2013). Understanding the rhythm of breathing: so near, yet so far. *Annual Review Of Physiology*, *75*, 423–452. 10.1146/annurev-physiol-040510-130049 23121137 10.1146/annurev-physiol-040510-130049 PMC3671763

[20] Ficker, D. M., So, E. L., Shen, W. K., Annegers, J. F., O’Brien, P. C., Cascino, G. D., & Belau, P. G. (1998). Population-based study of the incidence of sudden unexplained death in epilepsy. *Neurology*, *51*(5), 1270–1274. 10.1212/wnl.51.5.1270 9818844 10.1212/wnl.51.5.1270

[21] Fiore, G., de Tisi, J., O’Keeffe, A., Miserocchi, A., McEvoy, A. W., Sander, J. W., & Duncan, J. S. (2025). Long-term survival after adult epilepsy surgery: Mortality and predictors in a large cohort. *Epilepsia*, *66*(11), 4198–4210. 10.1111/epi.18564 40711417 10.1111/epi.18564 PMC12661268

[22] Forsgren, L., Hauser, W. A., Olafsson, E., Sander, J. W., Sillanpaa, M., & Tomson, T. (2005). Mortality of epilepsy in developed countries: a review. *Epilepsia 46 Suppl*, *11*, 18–27. 10.1111/j.1528-1167.2005.00403.x 10.1111/j.1528-1167.2005.00403.x 16393174

[23] Granbichler, C. A., Nashef, L., Selway, R., & Polkey, C. E. (2015). Mortality and SUDEP in epilepsy patients treated with vagus nerve stimulation. *Epilepsia*, *56*(2), 291–296. 10.1111/epi.12888 25580645 10.1111/epi.12888

[24] Gravitis, A. C., Wennberg, R., Carlen, P. L., Chinvarun, Y., Lira, V., Laze, J., Devinsky, O., & Bardakjian, B. L. (2025). Cardiorespiratory cross-frequency coupling biomarker for sudden unexpected death in epilepsy. *Epilepsia*. 10.1002/epi.70057 41389016 10.1002/epi.70057 PMC13075621

[25] Gray, P. A., Janczewski, W. A., Mellen, N., McCrimmon, D. R., & Feldman, J. L. (2001). Normal breathing requires preBotzinger complex neurokinin-1 receptor-expressing neurons. *Nature Neuroscience*, *4*(9), 927–930. 10.1038/nn0901-927 11528424 10.1038/nn0901-927 PMC2810393

[26] Gronborg, S., & Uldall, P. (2014). Mortality and causes of death in children referred to a tertiary epilepsy center. *European Journal Of Paediatric Neurology : Ejpn : Official Journal Of The European Paediatric Neurology Society*, *18*(1), 66–71. 10.1016/j.ejpn.2013.08.004 24100171 10.1016/j.ejpn.2013.08.004

[27] Grundmann, A., Brolly, J., Craig, D. P., Osland, K., Hanna, J., Hughes, E., Kerr, M. P., Donovan, B., & Thomas, R. H. (2025). The epilepsy deaths register: Third-party reports of SUDEP in adults and older adolescents. *Seizure*, *132*, 20–29. 10.1016/j.seizure.2025.08.031 40886616 10.1016/j.seizure.2025.08.031

[28] Han, Z., Chen, C., Christiansen, A., Ji, S., Lin, Q., Anumonwo, C., Liu, C., Leiser, S. C., Meena, Aznarez, I., Liau, G., & Isom, L. L. (2020). Antisense oligonucleotides increase Scn1a expression and reduce seizures and SUDEP incidence in a mouse model of Dravet syndrome. *Science Translational Medicine*, *12*(558). 10.1126/scitranslmed.aaz6100 10.1126/scitranslmed.aaz6100 32848094

[29] Harden, C., Tomson, T., Gloss, D., Buchhalter, J., Cross, J. H., Donner, E., French, J. A., Gil-Nagel, A., Hesdorffer, D. C., Smithson, W. H., Spitz, M. C., Walczak, T. S., Sander, J. W., & Ryvlin, P. (2017). Practice guideline summary: Sudden unexpected death in epilepsy incidence rates and risk factors: Report of the Guideline Development, Dissemination, and Implementation Subcommittee of the American Academy of Neurology and the American Epilepsy Society. *Neurology*, *88*(17), 1674–1680. 10.1212/WNL.0000000000003685 28438841 10.1212/WNL.0000000000003685

[30] Hesdorffer, D. C., Tomson, T., Benn, E., Sander, J. W., Nilsson, L., Langan, Y., Walczak, T. S., Beghi, E., Brodie, M. J., Hauser, A., & Epidemiology, I. C. (2011). Combined analysis of risk factors for SUDEP. *Epilepsia*, *52*(6), 1150–1159. 10.1111/j.1528-1167.2010.02952.x. o., & Subcommission on. 21671925 10.1111/j.1528-1167.2010.02952.x

[31] Hunanyan, A. S., Verma, A., Bidzimou, M. T., Biswas, D. D., Da Cruz, E., Srour, M. K., Marek, J., Hume, C., Elmallah, M. K., Landstrom, A. P., & Mikati, M. A. (2025). Progressive central cardiorespiratory rate downregulation and intensifying epilepsy lead to sudden unexpected death in epilepsy in mouse model of the most common human ATP1A3 mutation. *Epilepsia*, *66*(3), 899–913. 10.1111/epi.18236 39797721 10.1111/epi.18236 PMC11908895

[32] Kang, J. Y., Rabiei, A. H., Myint, L., & Nei, M. (2017). Equivocal significance of post-ictal generalized EEG suppression as a marker of SUDEP risk. *Seizure*, *48*, 28–32. 10.1016/j.seizure.2017.03.017 28380395 10.1016/j.seizure.2017.03.017

[33] Keller, A. E., Whitney, R., Li, S. A., Pollanen, M. S., & Donner, E. J. (2018). Incidence of sudden unexpected death in epilepsy in children is similar to adults. *Neurology*, *91*(2), e107–e111. 10.1212/WNL.0000000000005762 29884734 10.1212/WNL.0000000000005762

[34] Keller, A. E., Ho, J., Whitney, R., Li, S. A., Williams, A. S., Pollanen, M. S., & Donner, E. J. (2021). Autopsy-reported cause of death in a population-based cohort of sudden unexpected death in epilepsy. *Epilepsia*, *62*(2), 472–480. 10.1111/epi.16793 33400291 10.1111/epi.16793

[35] Klovgaard, M., Lynge, T. H., Tsiropoulos, I., Uldall, P. V., Banner, J., Winkel, B. G., Ryvlin, P., Tfelt-Hansen, J., & Sabers, A. (2021). Sudden unexpected death in epilepsy in persons younger than 50 years: A retrospective nationwide cohort study in Denmark. *Epilepsia*, *62*(10), 2405–2415. 10.1111/epi.17037 34418071 10.1111/epi.17037

[36] Langan, Y., Nashef, L., & Sander, J. W. (2005). Case-control study of SUDEP. *Neurology*, *64*(7), 1131–1133. 10.1212/01.WNL.0000156352.61328.CB 15824334 10.1212/01.WNL.0000156352.61328.CB

[37] Latreille, V., Abdennadher, M., Dworetzky, B. A., Ramel, J., White, D., Katz, E., Zarowski, M., Kothare, S., & Pavlova, M. (2017). Nocturnal seizures are associated with more severe hypoxemia and increased risk of postictal generalized EEG suppression. *Epilepsia*, *58*(9), e127–e131. 10.1111/epi.13841 28714130 10.1111/epi.13841 PMC5784438

[38] Lhatoo, S. D., Faulkner, H. J., Dembny, K., Trippick, K., Johnson, C., & Bird, J. M. (2010). An electroclinical case-control study of sudden unexpected death in epilepsy. *Annals Of Neurology*, *68*(6), 787–796. 10.1002/ana.22101 20882604 10.1002/ana.22101

[39] Lhatoo, S. D., Nei, M., Raghavan, M., Sperling, M., Zonjy, B., Lacuey, N., & Devinsky, O. (2016). Nonseizure SUDEP: Sudden unexpected death in epilepsy without preceding epileptic seizures. *Epilepsia*, *57*(7), 1161–1168. 10.1111/epi.13419 27221596 10.1111/epi.13419 PMC5541994

[40] Lin, Z., Si, Q., & Xiaoyi, Z. (2017). Obstructive sleep apnoea in patients with epilepsy: a meta-analysis. *Sleep & Breathing = Schlaf & Atmung*, *21*(2), 263–270. 10.1007/s11325-016-1391-3 27473532 10.1007/s11325-016-1391-3

[41] Lucchesi, M., Silverman, J. B., Sundaram, K., Kollmar, R., & Stewart, M. (2020). Proposed Mechanism-Based Risk Stratification and Algorithm to Prevent Sudden Death in Epilepsy. *Frontiers In Neurology*, *11*, 618859. 10.3389/fneur.2020.618859 33569036 10.3389/fneur.2020.618859 PMC7868441

[42] Magana-Tellez, O., Maganti, R., Hupp, N. J., Luo, X., Rani, S., Hampson, J. P., Ochoa-Urrea, M., Tallavajhula, S. S., Sainju, R. K., Friedman, D., Nei, M., Gehlbach, B. K., Schuele, S., Harper, R. M., Diehl, B., Bateman, L. M., Devinsky, O., Richerson, G. B., Lhatoo, S. D., & Lacuey, N. (2025). Sleep EEG and respiratory biomarkers of sudden unexpected death in epilepsy (SUDEP): a case-control study. *Lancet Neurology*, *24*(10), 840–849. 10.1016/S1474-4422(25)00273-X 40975100 10.1016/S1474-4422(25)00273-X PMC12707159

[43] Maguire, M. J., Jackson, C. F., Marson, A. G., & Nolan, S. J. (2016). Treatments for the prevention of Sudden Unexpected Death in Epilepsy (SUDEP). *Cochrane Database Systematic Review*, *7*(7), CD011792. 10.1002/14651858.CD011792.pub2 10.1002/14651858.CD011792.pub2 PMC6458047 27434597

[44] Mahr, K., Bergmann, M. P., Kay, L., Moller, L., Reif, P. S., Willems, L. M., Menzler, K., Schubert-Bast, S., Klein, K. M., Knake, S., Rosenow, F., Zollner, J. P., & Strzelczyk, A. (2020). Prone, lateral, or supine positioning at seizure onset determines the postictal body position: A multicenter video-EEG monitoring cohort study. *Seizure*, *76*, 173–178. 10.1016/j.seizure.2020.02.008 32109735 10.1016/j.seizure.2020.02.008

[45] Makowski, C., Wagner, M., & Haberlandt, E. (2024). „Sudden unexpected death in epilepsy (SUDEP) im Kindesalter – Auftreten im Zusammenhang mit selbstlimitierender Epilepsie mit zentrotemporalen Spikes (SeLECTS). *Clinical Epileptology*, *37*(1), 16–20. 10.1007/s10309-023-00645-6

[46] Maltseva, M., Rosenow, F., Schubert-Bast, S., Flege, S., Wolff, M., von Spiczak, S., Trollmann, R., Syrbe, S., Ruf, S., Polster, T., Neubauer, B. A., Mayer, T., Jacobs, J., Kurlemann, G., Kluger, G., Klotz, K. A., Kieslich, M., Kay, L., Hornemann, F., & Strzelczyk, A. (2024). Critical incidents, nocturnal supervision, and caregiver knowledge on SUDEP in patients with Dravet syndrome: A prospective multicenter study in Germany. *Epilepsia*, *65*(1), 115–126. 10.1111/epi.17799 37846648 10.1111/epi.17799

[47] Meletti, S., Burani, M., Ballerini, A., Giovannini, G., Micalizzi, E., Orlandi, N., Taruffi, L., Biagioli, N., Scolastico, S., Madrassi, L., Pugnaghi, M., & Vaudano, A. E. (2025). Persistent Postictal Central Apnea in Focal Seizures: Incidence, Features, and Imaging Findings. *Neurology*, *105*(4), e213856. 10.1212/WNL.0000000000213856 40694793 10.1212/WNL.0000000000213856 PMC12288844

[48] Nashef, L., So, E. L., Ryvlin, P., & Tomson, T. (2012). Unifying the definitions of sudden unexpected death in epilepsy. *Epilepsia*, *53*(2), 227–233. 10.1111/j.1528-1167.2011.03358.x 22191982 10.1111/j.1528-1167.2011.03358.x

[49] Ochoa-Urrea, M., Luo, X., Vilella, L., Lacuey, N., Omidi, S. J., Hupp, N. J., Talavera, B., Hampson, J. P., Rani, M. R. S., Tao, S., Li, X., Miyake, C. Y., Cui, L., Hampson, J. S., Chaitanya, G., Vakilna, Y. S., Sainju, R. K., Friedman, D., Nei, M., & Lhatoo, S. D. (2025). Risk markers for sudden unexpected death in epilepsy: an observational, prospective, multicentre cohort study. *Lancet*, *406*(10511), 1497–1507. 10.1016/S0140-6736(25)01636-8 40975113 10.1016/S0140-6736(25)01636-8 PMC12707170

[50] Panelli, R. J., & O’Brien, T. J. (2019). Epilepsy and seizure-related deaths: Mortality statistics do not tell the complete story. *Epilepsy Behav 98(Pt A)*, 266–272. 10.1016/j.yebeh.2019.07.003 10.1016/j.yebeh.2019.07.003 31408827

[51] Peng, W., Danison, J. L., & Seyal, M. (2017). Postictal generalized EEG suppression and respiratory dysfunction following generalized tonic-clonic seizures in sleep and wakefulness. *Epilepsia*, *58*(8), 1409–1414. 10.1111/epi.13805 28555759 10.1111/epi.13805

[52] Phabphal, K., Koonalintip, P., Sithinamsuwan, P., Wongsritrang, K., Amornpojnimman, T., Ekpitakdamrong, N., & Geater, A. F. (2021). Obstructive sleep apnea and sudden unexpected death in epilepsy in unselected patients with epilepsy: are they associated? *Sleep & Breathing = Schlaf & Atmung*, *25*(4), 1919–1924. 10.1007/s11325-021-02307-1 33580841 10.1007/s11325-021-02307-1

[53] Purnell, B. S., Braun, A., Fedele, D., Murugan, M., & Boison, D. (2022). Diaphragmatic pacing for the prevention of sudden unexpected death in epilepsy. *Brain Commun*, *4*(5), fcac232. 10.1093/braincomms/fcac232 36196086 10.1093/braincomms/fcac232 PMC9525001

[54] Purnell, B. S., Petrucci, A. N., Li, R., & Buchanan, G. F. (2024). Effect of adenosinergic manipulations on amygdala-kindled seizures in mice: Implications for sudden unexpected death in epilepsy. *Epilepsia*, *65*(9), 2812–2826. 10.1111/epi.18059 38980980 10.1111/epi.18059 PMC11534534

[55] Rheims, S., Chorfa, F., Michel, V., Hirsch, E., Maillard, L., Valton, L., Bartolomei, F., Derambure, P., Navarro, V., Biberon, J., Crespel, A., Nica, A., Martin, M. L., Mazzola, L., Petit, J., Rossero, V., Boulogne, S., Leclercq, M., Bezin, L., & Group, E. S. (2025). Efficacy of naloxone in reducing hypoxemia and duration of immobility following focal to bilateral tonic-clonic seizures. *Epilepsia Open*, *10*(3), 880–893. 10.1002/epi4.70046 40290094 10.1002/epi4.70046 PMC12163546

[56] Roliz, A. H., & Kothare, S. (2022). The Interaction Between Sleep and Epilepsy. *Current Neurology And Neuroscience Reports*, *22*(9), 551–563. 10.1007/s11910-022-01219-1 35802300 10.1007/s11910-022-01219-1

[57] Ruiz Vargas, E., Soros, P., Shoemaker, J. K., & Hachinski, V. (2016). Human cerebral circuitry related to cardiac control: A neuroimaging meta-analysis. *Annals Of Neurology*, *79*(5), 709–716. 10.1002/ana.24642 30240034 10.1002/ana.24642

[58] Ryvlin, P., Cucherat, M., & Rheims, S. (2011). Risk of sudden unexpected death in epilepsy in patients given adjunctive antiepileptic treatment for refractory seizures: a meta-analysis of placebo-controlled randomised trials. *Lancet Neurology*, *10*(11), 961–968. 10.1016/S1474-4422(11)70193-4 21937278 10.1016/S1474-4422(11)70193-4

[59] Ryvlin, P., Nashef, L., Lhatoo, S. D., Bateman, L. M., Bird, J., Bleasel, A., Boon, P., Crespel, A., Dworetzky, B. A., Hogenhaven, H., Lerche, H., Maillard, L., Malter, M. P., Marchal, C., Murthy, J. M., Nitsche, M., Pataraia, E., Rabben, T., Rheims, S., & Tomson, T. (2013). Incidence and mechanisms of cardiorespiratory arrests in epilepsy monitoring units (MORTEMUS): a retrospective study. *Lancet Neurology*, *12*(10), 966–977. 10.1016/S1474-4422(13)70214-X 24012372 10.1016/S1474-4422(13)70214-X

[60] Ryvlin, P., So, E. L., Gordon, C. M., Hesdorffer, D. C., Sperling, M. R., Devinsky, O., Bunker, M. T., Olin, B., & Friedman, D. (2018). Long-term surveillance of SUDEP in drug-resistant epilepsy patients treated with VNS therapy. *Epilepsia*, *59*(3), 562–572. 10.1111/epi.14002 29336017 10.1111/epi.14002

[61] Ryvlin, P., Huot, M., Valton, L., Maillard, L., Bartolomei, F., Derambure, P., Hirsch, E., Michel, V., Chassoux, F., Petit, J., Crespel, A., Biraben, A., Navarro, V., Kahane, P., De Toffol, B., Thomas, P., Rosenberg, S., Bernini, A., & Charlois, A. L. (2026). . group, R. M. s. Seizure-related biomarkers of sudden unexpected death in epilepsy (SUDEP) in drug-resistant focal epilepsy (REPO(2)MSE): a prospective, multicentre case-control study. *Lancet Neurol*, *25*(1), 50–60. 10.1016/S1474-4422(25)00379-5 10.1016/S1474-4422(25)00379-5 41285145

[62] Sainju, R. K., Dragon, D. N., Winnike, H. B., Vilella, L., Li, X., Lhatoo, S., Eyck, T., Wendt, P., Richerson, L. H., G. B., & Gehlbach, B. K. (2023). Interictal respiratory variability predicts severity of hypoxemia after generalized convulsive seizures. *Epilepsia*, *64*(9), 2373–2384. 10.1111/epi.17691 37344924 10.1111/epi.17691 PMC10538446

[63] Schubert-Bast, S., Kay, L., Simon, A., Wyatt, G., Holland, R., Rosenow, F., & Strzelczyk, A. (2022). Epidemiology, healthcare resource use, and mortality in patients with probable Dravet syndrome: A population-based study on German health insurance data. *Epilepsy & Behavior*, *126*, 108442. 10.1016/j.yebeh.2021.108442 34864381 10.1016/j.yebeh.2021.108442

[64] Schwab, C., Wadle, N. E., Knake, S., von Podewils, F., Siebenbrodt, K., Kohlhase, K., Schulz, J., Menzler, K., Mann, C., Rosenow, F., Seifart, C., & Strzelczyk, A. (2021). Patients’ knowledge about epilepsy-related risks, morbidity, and mortality: A multicenter cohort study from Germany. *Epilepsy & Behavior*, *124*, 108343. 10.1016/j.yebeh.2021.108343 34619541 10.1016/j.yebeh.2021.108343

[65] Seymour, N., Granbichler, C. A., Polkey, C. E., & Nashef, L. (2012). Mortality after temporal lobe epilepsy surgery. *Epilepsia*, *53*(2), 267–271. 10.1111/j.1528-1167.2011.03343.x 22126418 10.1111/j.1528-1167.2011.03343.x

[66] Sillanpaa, M., & Shinnar, S. (2010). Long-term mortality in childhood-onset epilepsy. *New England Journal Of Medicine*, *363*(26), 2522–2529. 10.1056/NEJMoa0911610 21175314 10.1056/NEJMoa0911610

[67] Singh, K., Katz, E. S., Zarowski, M., Loddenkemper, T., Llewellyn, N., Manganaro, S., Gregas, M., Pavlova, M., & Kothare, S. V. (2013). Cardiopulmonary complications during pediatric seizures: a prelude to understanding SUDEP. *Epilepsia*, *54*(6), 1083–1091. 10.1111/epi.12153 23731396 10.1111/epi.12153 PMC5304951

[68] Sivathamboo, S., Friedman, D., Laze, J., Nightscales, R., Chen, Z., Kuhlmann, L., Devore, S., Macefield, V., Kwan, P., D’Souza, W., Berkovic, S. F., Perucca, P., O’Brien, T. J., Devinsky, O., & Group, M. S. B. S. (2021). Association of Short-term Heart Rate Variability and Sudden Unexpected Death in Epilepsy. *Neurology*, *97*(24), e2357–e2367. 10.1212/WNL.0000000000012946 34649884 10.1212/WNL.0000000000012946

[69] Smith, J. C., Ellenberger, H. H., Ballanyi, K., Richter, D. W., & Feldman, J. L. (1991). Pre-Botzinger complex: a brainstem region that may generate respiratory rhythm in mammals. *Science*, *254*(5032), 726–729. 10.1126/science.1683005 1683005 10.1126/science.1683005 PMC3209964

[70] Sourbron, J., & Lagae, L. (2022). Serotonin receptors in epilepsy: Novel treatment targets? *Epilepsia Open*, *7*(2), 231–246. 10.1002/epi4.12580 35075810 10.1002/epi4.12580 PMC9159250

[71] Sperling, M. R., Rosenfeld, W. E., Watson, J., & Klein, P. (2025). Seizure freedom and reducing the risk of sudden unexpected death in patients with focal epilepsy treated with cenobamate or other antiseizure medications. *Epilepsia 66 Suppl*, *1*(Suppl 1), 4–14. 10.1111/epi.18307 10.1111/epi.18307 PMC11922000 40105710

[72] Strzelczyk, A., Zschebek, G., Bauer, S., Baumgartner, C., Grond, M., Hermsen, A., Kieslich, M., Kramer, G., Kurlemann, G., May, T. W., Mayer, T., Neubauer, B. A., Pfafflin, M., Plecko, B., Ryvlin, P., Schubert-Bast, S., Stefan, H., Trinka, E., Knake, S., & Rosenow, F. (2016). Predictors of and attitudes toward counseling about SUDEP and other epilepsy risk factors among Austrian, German, and Swiss neurologists and neuropediatricians. *Epilepsia*, *57*(4), 612–620. 10.1111/epi.13337 26899504 10.1111/epi.13337

[73] Surges, R., Strzelczyk, A., Scott, C. A., Walker, M. C., & Sander, J. W. (2011). Postictal generalized electroencephalographic suppression is associated with generalized seizures. *Epilepsy & Behavior*, *21*(3), 271–274. 10.1016/j.yebeh.2011.04.008 21570920 10.1016/j.yebeh.2011.04.008

[74] Sveinsson, O., Andersson, T., Carlsson, S., & Tomson, T. (2017). The incidence of SUDEP: A nationwide population-based cohort study. *Neurology*, *89*(2), 170–177. 10.1212/WNL.0000000000004094 28592455 10.1212/WNL.0000000000004094

[75] Sveinsson, O., Andersson, T., Carlsson, S., & Tomson, T. (2018). Circumstances of SUDEP: A nationwide population-based case series. *Epilepsia*, *59*(5), 1074–1082. 10.1111/epi.14079 29663344 10.1111/epi.14079

[76] Sveinsson, O., Andersson, T., Mattsson, P., Carlsson, S., & Tomson, T. (2020). Clinical risk factors in SUDEP: A nationwide population-based case-control study. *Neurology*, *94*(4), e419–e429. 10.1212/WNL.0000000000008741 31831600 10.1212/WNL.0000000000008741 PMC7079690

[77] Sveinsson, O., Andersson, T., Carlsson, S., & Tomson, T. (2023). Type, Etiology, and Duration of Epilepsy as Risk Factors for SUDEP: Further Analyses of a Population-Based Case-Control Study. *Neurology*, *101*(22), e2257–e2265. 10.1212/WNL.0000000000207921 37813583 10.1212/WNL.0000000000207921 PMC10727222

[78] Swor, D., Juneja, P., Constantine, C., Mann, C., Rosenow, F., & LaRoche, S. (2024). Management of status epilepticus in pregnancy: a clinician survey. *Neurol Res Pract*, *6*(1), 3. 10.1186/s42466-023-00295-z 38233889 10.1186/s42466-023-00295-z PMC10795404

[79] Tarighati Rasekhi, R., Devlin, K. N., Mass, J. A., Donmez, M., Asma, B., Sperling, M. R., & Nei, M. (2021). Improving prediction of sudden unexpected death in epilepsy: From SUDEP-7 to SUDEP-3. *Epilepsia*, *62*(7), 1536–1545. 10.1111/epi.16928 34086290 10.1111/epi.16928

[80] Thurman, D. J., Hesdorffer, D. C., & French, J. A. (2014). Sudden unexpected death in epilepsy: assessing the public health burden. *Epilepsia*, *55*(10), 1479–1485. 10.1111/epi.12666 24903551 10.1111/epi.12666

[81] Tian, X., Wang, J., Wang, X., Yang, X., Li, T., Deng, J., Tang, C., Wang, M., & Luan, G. (2025). Topography of the insular cortex in heart rate control: high-precision mapping reveals critical role of the middle short gyrus. *Front Neurosci*, *19*, 1665378. 10.3389/fnins.2025.1665378 41040956 10.3389/fnins.2025.1665378 PMC12484038

[82] Tomson, T., Sveinsson, O., Carlsson, S., & Andersson, T. (2018). Evolution over time of SUDEP incidence: A nationwide population-based cohort study. *Epilepsia*, *59*(8), e120–e124. 10.1111/epi.14460 29905938 10.1111/epi.14460

[83] Tomson, T., Andersson, T., Carlsson, S., & Sveinsson, O. (2025). Influence of Risk Factor Combinations on Incidence Rates of SUDEP: A Population-Based Study. *Neurology*, *104*(5), e213372. 10.1212/WNL.0000000000213372 39908470 10.1212/WNL.0000000000213372

[84] Tononi, G., & Cirelli, C. (2006). Sleep function and synaptic homeostasis. *Sleep Medicine Reviews*, *10*(1), 49–62. 10.1016/j.smrv.2005.05.002 16376591 10.1016/j.smrv.2005.05.002

[85] Vilella, L., Miyake, C. Y., Chaitanya, G., Hampson, J. P., Omidi, S. J., Ochoa-Urrea, M., Talavera, B., Mancera, O., Hupp, N. J., Hampson, J. S., Rani, M. R. S., Lacuey, N., Tao, S., Sainju, R. K., Friedman, D., Nei, M., Scott, C. A., Gehlbach, B., Schuele, S. U., & Lhatoo, S. D. (2024). Incidence and Types of Cardiac Arrhythmias in the Peri-Ictal Period in Patients Having a Generalized Convulsive Seizure. *Neurology*, *103*(1), e209501. 10.1212/WNL.0000000000209501 38870452 10.1212/WNL.0000000000209501 PMC11759939

[86] Wadle, N. E., Schwab, C., Seifart, C., von Podewils, F., Knake, S., Willems, L. M., Menzler, K., Schulz, J., Conradi, N., Rosenow, F., & Strzelczyk, A. (2023). Prospective, longitudinal, multicenter study on the provision of information regarding sudden unexpected death in epilepsy to adults with epilepsy. *Epilepsia*, *64*(2), 406–419. 10.1111/epi.17481 36546828 10.1111/epi.17481

[87] Whitney, R., Keller, A., Li, S. A., Datta, A. N., MacDonald, M., Nabavi Nouri, M., Pohl, D., Sell, E., Ronen, G. M., Sidhu, M., Simard-Tremblay, E., Pollanen, M. S., & Donner, E. J. (2025). Circumstances surrounding sudden unexpected death in epilepsy in children: A national case series. *Epilepsia*, *66*(6), 1988–2000. 10.1111/epi.18339 40186506 10.1111/epi.18339

[88] Zeicu, C., Legouhy, A., Scott, C. A., Oliveira, J. F. A., Winston, G. P., Duncan, J. S., Vos, S. B., Thom, M., Lhatoo, S., Zhang, H., Harper, R. M., & Diehl, B. (2023). Altered amygdala volumes and microstructure in focal epilepsy patients with tonic-clonic seizures, ictal, and post-convulsive central apnea. *Epilepsia*, *64*(12), 3307–3318. 10.1111/epi.17804 37857465 10.1111/epi.17804 PMC10952501

[89] Zollner, J. P., Mann, C., Willems, L., von Podewils, F., Langenbruch, L., Bierhansl, L., Knake, S., Menzler, K., Schulz, J., Gaida, B., Rosenow, F., & Strzelczyk, A. (2025). Sleep quality and its correlates in people with epilepsy: A multicenter cross-sectional study in Germany. *Epilepsia*, *66*(12), 4764–4779. 10.1111/epi.18599 40844390 10.1111/epi.18599 PMC12779309

